# Optimization of Location-Routing Problem in Emergency Logistics Considering Carbon Emissions

**DOI:** 10.3390/ijerph16162982

**Published:** 2019-08-19

**Authors:** Ling Shen, Fengming Tao, Yuhe Shi, Ruiru Qin

**Affiliations:** 1 College of Mechanical Engineering, Chongqing University, Chongqing 400044, China; 2 School of Management Science and Real Estate, Chongqing University, Chongqing 400044, China; 3 School of Transportation and Logistics, Southwest Jiaotong University, Chengdu 610031, China; 4 School of Aeronautic Science and Engineering, Beihang University, Beijing 100191, China

**Keywords:** emergency logistics, location routing problem, environment effect, two-phase algorithm

## Abstract

In order to solve the optimization problem of emergency logistics system, this paper provides an environmental protection point of view and combines with the overall optimization idea of emergency logistics system, where a fuzzy low-carbon open location-routing problem (FLCOLRP) model in emergency logistics is constructed with the multi-objective function, which includes the minimum delivery time, total costs and carbon emissions. Taking into account the uncertainty of the needs of the disaster area, this article illustrates a triangular fuzzy function to gain fuzzy requirements. This model is tackled by a hybrid two-stage algorithm: Particle swarm optimization is adopted to obtain the initial optimal solution, which is further optimized by tabu search, due to its global optimization capability. The effectiveness of the proposed algorithm is verified by the classic database in LRP. What’s more, an example of a post-earthquake rescue is used in the model for acquiring reliable conclusions, and the application of the model is tested by setting different target weight values. According to these results, some constructive proposals are propounded for the government to manage emergency logistics and for the public to aware and measure environmental emergency after disasters.

## 1. Introduction

Since the 1950s, the number and scale of major natural disasters (such as earthquakes, tsunamis, cyclones, floods, volcanoes and etc.) have grown exponentially [1]. These have resulted in catastrophic consequences, which are closely related to resource waste and environmental degradation. In 1991, Tropical Storm Bangladesh caused flooding and resulted in nearly 140,000 casualties. In 2004, the Indian Ocean tsunami caused 230,000 deaths. In 2008, the Wenchuan earthquake in China killed 69,227 people and injured 374,643 people. Moreover, in August 2017, Hurricane Harvey produced $180 billion in damage and impacted 13 million people. Nevertheless, the occurrence of natural disasters is inevitable [2]. In the face of frequent natural disasters, taking active measures has become a major problem that all countries in the world must face in solving sudden natural disasters [3].

In order to reduce severity and fatality in a disaster, emergency logistics has been an efficient and effective relief means [4,5], which contents a series of actions performed before, during and after a natural disaster. Compared with traditional commercial logistics, emergency logistics is more challenging and unpredictable [6]. The two cores of the relief material dispatching decision are the location of emergency distribution centers (DCs) before disasters and the route arrangement of vehicles after disasters, which are called location allocation problems (LAP) and vehicle routing problems (VRP) respectively. On the one hand, it is necessary to establish a certain number and scale of emergency DCs in suitable locations to store and allocate relief materials from all over the country; on the other hand, it is necessary to dispatch appropriate vehicles to select reasonable routes to distribute relief materials from the DCs to the demand points. In actual life, LAP and VRP are highly correlated, of which the combination is called location routing problems (LRP). However, when distributing relief supplies, the vehicles emit a certain amount of carbon dioxide, which will exacerbate climate change [7]. The deterioration of the environment is one of the considerable causes of frequent natural disasters. Thus, it is essential to take carbon emissions into account, while optimizing location routing problem in emergency logistics, for realizing a relative balance between rescue activities and environmental protection in the long run.

The remainder of this paper is organized as follows. In the next section, the previous literature on LRP is introduced. Section 3 builds a multi-objective FLCOLRP model. A two-phase algorithm is proposed to solve the built model in Section 4. Section 5 performs a series of experiments and analyzes the results. Finally, the conclusions of this article and the outlook for future research are presented in Section 6.

## 2. Literature Review

Since the core of this research is to gain an overall optimal solution for LRP in emergency logistics considering carbon emissions. Thus, we will review the related literature from the following two aspects: The algorithm of LRP in emergency logistics and sustainability issues in LRP, including carbon emissions.

### 2.1. Algorithm for LRP in Emergency Logistics

Due to the high complexity and difficulty of LAP and VRP [8,9,10], they were presented separately in previous studies. Actually, the interaction between LAP and VRP was put forward in the 1970s [11]. In later research, LAP and VRP started to be integrated as a complex combinatorial optimization problem called LRP. There are various exact methods, approximation algorithms and heuristic algorithms proposed to solve LRP [12], such as the branch-and-price method [13], the Lagrangian Relaxation-Granular Tabu Search (LRGTS) algorithm [14], the Multiple Ant Colony Optimization (MACO) algorithm [15], the Non-dominated Sorting Genetic Algorithm II (NSGA-II) [16], the Particle Swarm Optimization (PSO) algorithm [17], the integrated Non-dominated Sorting Genetic Algorithm II (NSGA-II) and Multi-Objective Particle Swarm Optimization (MOPSO) [18] and etc.

LRP has plenty of real-life applications [19] involving newspaper distribution, energy enterprise material distribution, e-commerce distribution, garbage recycling, military application and etc. Over the last decade, some scholars paid attention to LRP in emergency logistics owing to the active occurrence and the devastating aftermaths of natural disasters. Yi and Özdamar [20] discussed the coordination of material distribution and wounded migration in disaster response activities and built a mixed-integer multi-commodity network flow model which considered the location of temporary medical points, the assignment of medical personnel, the allocation of relief material and the decision of vehicle transportation path. In this study, a simple routing algorithm and a linear solution were proposed to solve the model. Zheng et al. [21], from the perspective of integration and optimization of emergency logistics system, established a multi-objective LRP optimization model with fuzzy demand for relief materials. In order to deal with the model, they introduced a new multi-objective genetic algorithm which averted the boundedness of traditional multi-objective optimization ways. Based on the research [21], Ma et al. [22] built a fuzzy multi-objective open LRP model which added the deadlines, the different types of delivery vehicles, as well as the open vehicle paths. A hybrid genetic algorithm combining heuristic rules was designed in this study. Rath and Gutjahr [23] presented the modified warehouse location-routing problem in disaster relief and established a multi-objective mixed-integer linear programming model which included strategic costs, operative costs and uncovered demand. An exact method was introduced to tackled small instances; A constraint pool heuristic was more suitable for larger examples. Both of them adopt the adaptive epsilon-constraint algorithm as the basic method. Alem et al. [24] addressed a novel two-phase stochastic network flow model which involved the preposition of disaster relief points and the decision for transportation. A simple two-phase heuristic was presented to solve this model and tested by real cases.

### 2.2. Sustainability Issues in LRP

With the increasing emphasis on the environment, a large number of articles on low-carbon transportation have emerged. In recent years, some studies have begun to concern about the reduction of carbon emissions in LRP. In 2014, Govindan et al. [25] designed a sustainable supply chain network (SCN) of perishable food and addressed a multi-objective optimization model to solve LRP with time windows. The economic objective of the model was to minimize the fixed costs, transportation costs, inventory costs, variable costs, penalty costs. The environmental objective of the model was to calculate greenhouse gas emissions throughout the network. In the same year, Ramos et al. [26] presented a sustainable reverse logistics system and proposed the three concerns: Economic aspects, environmental aspects and social aspects. This paper debated the balance between the objectives and employed a real recyclable waste collection case to gain an approximate value of the Pareto frontier. Tang et al. [27] established a location-routing-inventory integration problem optimization model, which introduced the carbon-capped difference. The objective of the model was to decline the total costs and carbon emissions. In this study, carbon trading prices were proposed to convert carbon emissions into carbon emissions costs in the objective function. Based on the research [27], Tang et al. [28] considered the customer’s limited “carbon behavior” preferences and introduced an environmental factor as a feature vector for carbon emissions in 2016. A multi-objective function with the lowest cost and the lowest carbon emissions was constructed. This paper proved that the customer’s limited “carbon behavior” preferences and the product environmental degree had an impact on the company’s operational plans and revenue levels. In 2017, a green capacitated location-routing problem was firstly addressed by Toro et al. [29]. They proposed the location of multi-depots and the routing of multi-vehicles, and developed a novel mathematical model which considered the greenhouse gas emissions. The target of this model was to minimize operating costs and minimize fuel consumption. In 2018, Ebrahimi [30] raised a sustainable loop-locked SCN and formulated a location-allocation-routing optimization model with the goals of minimizing the total costs and the environmental impact. In order to test the availability of the model, he adopted a real Iranian case. Meanwhile, Wang et al. [31] studied the low-carbon LRP for cold chain logistics and designed the mathematical model with the lowest total costs, including carbon emissions costs. In this study, it was proved that a certain range of carbon taxes could affect the carbon emissions of the cold chain logistics industry. Recently, Zhang et al. [32] introduced the sustainable LRP in emergency logistics and established a multi-objective model which included the targets of minimum travel time, relief costs, carbon emissions. They regarded travel time as their main goal and regard relief costs and carbon emissions as restrictions, converting multi-objective into single-objective.

To sum up, according to the analysis of the above two parts, there are numerous pieces of literature on the algorithm of LRP in emergency logistics. Simultaneously, the studies about sustainability issues in LRP have appeared extensively in the last five years. The objectives of the current studies are mainly to minimize the total costs and the environment effect. Taking into account the time urgency of emergency logistics, the study of LRP in emergency logistic should put travel time into model. Nonetheless, there are relatively few studies in which the LRP in emergency logistics and the development of sustainability are combined. This only related literature [32] studied the multi-depot emergency LRP with uncertainty theory tackled by a hybrid genetic algorithm and focused on verifying the effectiveness and robustness of the algorithm. Although the research considered travel time, distribution costs and environmental impacts into the model, further research on the low-carbon LRP in emergency logistics is necessary. In view of this, in this paper, we establish a fuzzy low-carbon open location-routing problem (FLCOLRP) model of emergency logistics and discuss the interconnection between the objective function of time, economic, as well as environment. A hybrid PSO-TS algorithm is designed to deal with the model in this study. What’s more, this paper employs the triangular fuzzy function tackling the unpredictability of demand in disaster areas.

## 3. Mathematical Model

### 3.1. Problem Description

**Figure 1:**
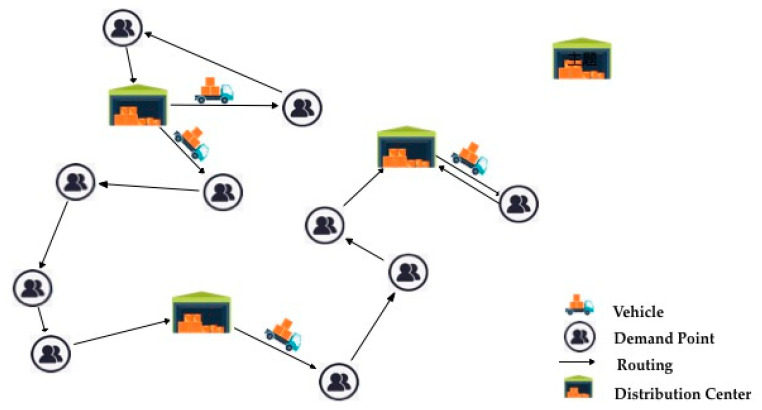
A simplified routing diagram of LRP.

The FLCOLRP model of emergency logistics studied in this paper can be stated as follows. There are some candidate distribution centers dispatching relief materials to the demand points by several different types of vehicles. The construction cost of each candidate distribution center and the operation cost of each vehicle are known, and the demand can be expressed by a triangular fuzzy function. After completing each delivery task, the vehicle may go to different distribution centers to continue the delivery task according to the need and finally return to the nearest distribution center. Under the constraints of time, space and resources, the multi-objective FLCOLRP model is established with the objectives of the lowest delivery time, total costs and carbon emissions, thus obtaining a fast, economical and environmentally friendly location-routing scheme and ensuring the distribution of relief materials simultaneously. Figure 1 shows a simplified diagram of the problem studied in this article.

### 3.2. Notations

Based on the needs to establish the model, Table 1 presents the corresponding notations applied in this paper.

### 3.3. Model Construction

The FLCOLRP model of relief materials distribution in this paper takes the delivery time minimum, the total costs minimum, as well as the carbon emissions minimum as the objective functions. Above all, we analyze the components of three objective functions separately, and then the specific formulation of the FLCOLRP model is determined by these components.

#### 3.3.1. Analysis of Objectives Function

1. Delivery Time

In emergency logistics, it is critical to delivering relief supplies from the distribution centers to the demand points as quickly as possible for reducing post-disaster loses. Thus, the minimization of delivery time is significant. In this paper, the delivery time T in the FLCOLRP model can be expressed as:

(1) $$T=\underset{h\in V_{h}}{\sum }\underset{i\in D_{c}\cup N_{d}}{\sum }\underset{j\in D_{c}\cup N_{d}}{\sum }t_{ij}x_{ij}^{h}.$$

2. Total Costs

(1) Fixed Costs

In the FLCOLRP model, the fixed costs are actually the construction costs of the distribution centers. However, due to some factors, such as land price levels and natural conditions, candidate distribution centers have different construction costs. The total fixed costs $C_{f}$ in the FLCOLRP model can be calculated as:

(2) $$C_{f}=\underset{i\in D_{c}}{\sum }\left(C_{i}y_{i}+\underset{h\in V_{h}}{\sum }\underset{m\in P_{m}}{\sum }\underset{i\in D_{c}\cup N_{d}}{\sum }H_{h}z_{mhi}^{c}\right).$$

(2) Transportation Costs

When the vehicle transports relief supplies from the distribution center to the demand point, it may encounter many situations, such as road damage, broken vehicles, extreme weather, etc. In this study, we only study research under normal conditions. The transportation costs $C_{t}$ in the FLCOLRP model can be expressed as:

(3) $$C_{t}=S_{1}\underset{h\in V_{h}}{\sum }\underset{i\in D_{c}\cup N_{d}}{\sum }\underset{j\in D_{c}\cup N_{d}}{\sum }d_{ij}x_{ij}^{h}.$$

(3) Penalty Costs

It is nearly impossible to gain the precise demand for relief supplies before the disaster, because the demand in the disaster area is fuzzy and uncertain. Hence, this paper utilizes the triangular fuzzy number and proposes the most optimistic demand, the most likely demand and the most pessimistic demand to describe the estimated demand $\tilde{ED_{i}}$ . The relationship of them is shown in the formulas below:

(4) $$\tilde{ED_{i}}=\langle {\left[ED_{i}\right]}^{o},{\left[ED_{i}\right]}^{l},{\left[ED_{i}\right]}^{p}\rangle ,$$

(5) $${\left[ED_{i}\right]}^{o}\leq {\left[ED_{i}\right]}^{l}\leq {\left[ED_{i}\right]}^{p}.$$

In addition, the weighted method can be adopted to convert the triangular fuzzy number into a certain value. Thus, the estimated demand $\tilde{ED_{i}}$ can be calculated with the equation:

(6) $$\tilde{ED_{i}}=\omega_{1}{\left[ED_{i}\right]}^{o}+\omega_{2}{\left[ED_{i}\right]}^{l}+\omega_{3}{\left[ED_{i}\right]}^{p}.$$

However, the number of relief materials transported by the vehicle to the demand point may be less than the estimated demand because of the limitation of the capacity of the vehicle and the distribution center. Therefore, when the demand for the demand point is not met, it will be given a certain penalty. The penalty costs of the FLCOLRP model can be expressed as:

(7) $$C_{q}=S_{2}\underset{c\in D_{c}}{\sum }\underset{h\in V_{h}}{\sum }\underset{m\in P_{m}}{\sum }\left(\underset{i\in N_{d}}{\sum }\tilde{ED_{i}}z_{mhi}^{c}-M_{h}\right).$$

3. Carbon emissions

According to statistics from the Environmental Protection Agency, the transportation sector produces the largest share of greenhouse gas emissions in the world [33]. The carbon emissions of transportation are linearly related to fuel consumption. But, the fuel consumption is affected by many factors, such as vehicle speed, vehicle load, road gradient, driving distance, etc. This paper refers to the literature [34] to calculate the fuel consumption $U_{c}\;$ , which can be shown in the formulas below:

(8) $$U_{c}=\underset{c\in D_{c}}{\sum }\underset{h\in V_{h}}{\sum }\underset{m\in P_{m}}{\sum }\underset{i\in D_{c}\cup N_{d}}{\sum }\underset{j\in D_{c}\cup N_{d}}{\sum }\left(\mu -\frac{\mu_{0}-\mu }{M_{h}}M_{ijh}\right)d_{ij}x_{ij}^{h}z_{mhi}^{c}.$$

Therefore, the carbon emissions $E_{co_{2}}$ of the FLCOLRP model can be expressed as:

(9) $$E_{co_{2}}=\tau U_{c}.$$

#### 3.3.2. FLCOLRP Model Setting

Based on the detailed analysis of the three objectives, the established multi-objective FLCOLRP model is shown as follows:

(10) $$MinF=\underset{h\in V_{h}}{\sum }\underset{i\in D_{c}\cup N_{d}}{\sum }\underset{j\in D_{c}\cup N_{d}}{\sum }t_{ij}x_{ij}^{h}$$

(11) $$MinC=\underset{i\in D_{h}}{\sum }(C_{i}y_{i}+\underset{h\in V_{h}}{\sum }\underset{m\in P_{m}}{\sum }\underset{i\in D_{c}\cup N_{d}}{\sum }H_{h}z_{mhi}^{c})+S_{1}\underset{h\in V_{h}}{\sum }\underset{i\in D_{c}\cup N_{d}}{\sum }\underset{j\in D_{c}\cup N_{d}}{\sum }d_{ij}x_{ij}^{h}+S_{2}\underset{c\in D_{c}}{\sum }\underset{h\in V_{h}}{\sum }\underset{m\in P_{m}}{\sum }\left(\underset{i\in N_{d}}{\sum }\tilde{ED_{i}}z_{mhi}^{c}-M_{h}\right)$$

(12) $$MinE_{co_{2}}=\tau \underset{c\in D_{c}}{\sum }\underset{h\in V_{h}}{\sum }\underset{m\in P_{m}}{\sum }\underset{i\in D_{c}\cup N_{d}}{\sum }\underset{j\in D_{c}\cup N_{d}}{\sum }\left(\mu -\frac{\mu_{0}-\mu }{M_{h}}M_{ijh}\right)d_{ij}x_{ij}^{h}z_{mhi}^{c}$$

Subject to:

(13) $$\underset{h\in V_{h}}{\sum }\underset{i\in D_{c}\cup N_{d}}{\sum }\underset{j\in D_{c}\cup N_{d}}{\sum }x_{ij}^{h}-y_{c}\geq 0,\;\forall c\in D_{o}\;$$

(14) $$\underset{i\in D_{c}\cup N_{d}}{\sum }\underset{j\in D_{c}\cup N_{d}}{\sum }x_{ij}^{h}-y_{c}\leq 0,\;\forall h\in V_{h},\forall c\in D_{c}⋂(C_{U}D_{o})$$

(15) $$\underset{h\in V_{h}}{\sum }\underset{i\in D_{c}\cup N_{d}}{\sum }x_{ij}^{h}=1,\forall j\in N_{d}$$

(16) $$\underset{h\in V_{h}}{\sum }\underset{m\in P_{m}}{\sum }\underset{i\in N_{d}}{\sum }\tilde{ED_{i}}z_{mhi}^{c}\leq W_{c},\forall c\in D_{c}$$

(17) $$\underset{i\in D_{c}\cup N_{d}}{\sum }\underset{j\in D_{c}\cup N_{d}}{\sum }d_{ij}x_{ij}^{h}z_{mhi}^{c}\leq O_{h},\forall h\in V_{h},\forall m\in SP_{m},\forall c\in D_{c}$$

(18) $$\underset{i\in D_{c}\cup N_{d}}{\sum }\underset{j\in D_{c}\cup N_{d}}{\sum }x_{ij}^{h}=1,\forall h\in V_{h}$$

(19) $$\underset{i\in N_{d}}{\sum }\underset{j\in D_{c}\cup N_{d}}{\sum }x_{ij}^{h}=1,\forall h\in V_{h}.$$

This model has three objective functions: The first objective (10) is to minimize the delivery time of relief supplies; the second objective (11) is to minimize the total costs of fixed costs, transportation costs and penalty costs for emergency logistics system; the third objective (12) is to minimize the carbon emissions. At least one vehicle is assigned for each open distribution center, and no vehicles can be assigned for unopened distribution centers, which are imposed by constraint (13) and constraint (14). Each demand point is only served once by one vehicle presented by Constraint (15). Constraint (16) addresses the amount delivered from each distribution center does not exceed the capacity of the distribution center. The distance traveled by each vehicle does not outstrip the limit, which is shown by constraint (17). Constraint (18) and constraint (19) illustrate vehicles must depart from the distribution center and can return to any distribution center. 

## 4. Design of PSO-TS Algorithm

**Figure 2:**
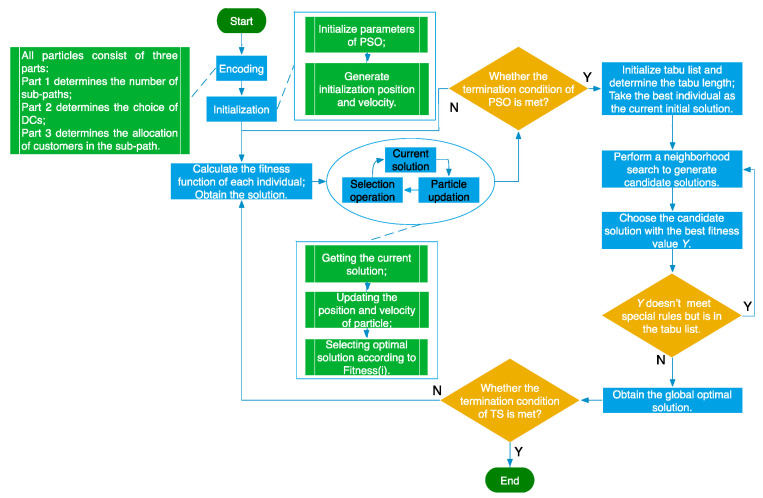
Basic process of PSO-TS.

LRP is an NP-hard problem [7,35], and a PSO algorithm combining Tabu Search (TS) is introduced to solve the model in this paper on account of the simplicity and flexibility of PSO algorithm and the ability of TS to obtain global optimal solutions. In the first stage, PSO algorithm is applied to obtain the partial optimization solution. In the second stage, TS is adopted to acquire the global optimization solution. The basic process of the hybrid algorithm is described in Figure 2.

Step 1: Particle coding. All particles are composed of three parts: Part 1 has *D* particles (*D* is the number of demand points), the value of each particle represents the sub-path number to which each demand point belongs and is randomly selected from the natural number of 1 to *A* ( $A=\sum_{i}^{N_{d}}\tilde{ED_{i}}/\min\left(M_{h}\right)$ ); Part 2 has *P* particles, the value of each particle represents the initial DC corresponding to each sub-path and is randomly selected from the natural number of 1 to *C* (*C* is the number of DCs); Part 3 has *D* particles, the value of each particle represents the order of the various demand points in each sub-path and is randomly selected from the natural number of *C* to *C + D*. Hence, the total length of coding is 2*D* + *A.* What’s more, the particles in the parts 1 and 3 correspond one-to-one, and the demand points in part 3 corresponding to the particles having the same value in part 1 are on the same path.

For example, there are 4 DCs (number 1 to 4) and 8 demand points (number 5 to 12), *A* = 5 (number 1 to 5). For the following particles:

As displayed in Table 2, part 1 includes 2, 3 and 4, indicating that there are three sub-paths. The positions corresponding to 2nd, 3rd and 4th in Part 2 are 2, 3 and 3 respectively, so DCs of 2 and 3 are established. It should be explained that the 1st and 5th of part 2 are numbered 1 and 4 respectively, but the DCs of 1 and 4 are not actually built. The 3rd and 7th of part 1 are numbered 2, and the corresponding positions in part 3 are the demand points 5 and 6, which show that these demand points are in the sub-path 1 and the driving order in sub-path 1 is 5-6. The 2nd in part 2 is numbered 2, representing that the starting point of sub-path 1 is distribution center 2—thus, the sub-path 1 is 2-5-6. In a similar way, the sub-path 2 is 10-8-9, and sub-path 3 is 7-12-11.

Step 2: Initializing parameters of PSO and generating the initialization position and velocity of the particle randomly.

Step 3: Determining the optimal solution by PSO. The built FLCOLRP model of this paper is a multi-objective optimization problem, which usually is solved by weighting methods, constraint methods, goal planning methods, and minimax methods [18]. Since the objective functions and constraints of the FLCOLRP model are linear functions, this paper utilizes the weighting method to deal with the multi-objective model. Thus, the fitness function of this paper is expressed as: $\mathrm{Fitness}\left(i\right)=w_{1}F\left(i\right)/F{\left(i\right)}^{min}+w_{2}C\left(i\right)/C{\left(i\right)}^{min}+w_{3}E_{co_{2}}\left(i\right)/E_{co_{2}}{\left(i\right)}^{min}$ , and $0<w_{1},\;w_{2},\;w_{3}<1,\;w_{1}+\;w_{2}+\;w_{3}=1,$ where $w_{1},\;w_{2}$ and $w_{3}$ are the weight of the objective function 1 , 2 and 3 respectively. In order to obtain the optimal solution, the position and velocity of particles need to be updated. Then, the optimal solution can be determined according to the value of the fitness function.

Step 4: Setting termination conditions of the PSO algorithm. The number of the population is $N_{p}.$ When the number of the iteration is greater than $N_{p}$ , the PSO algorithm ends, and the current optimal solution is regarded as the partial optimization solution.

Step 5: Initializing tabu list, determining tabu length and putting the partial optimization solution of PSO as the initial solution of TS.

Step 6: Determining the optimal solution by TS. First, according to the feature of the particle coding, this paper randomly selects the neighborhood search algorithm from the three routes (swap, reversion and insertion). Next, based on the special rules, tabu objects are defined, and tabu tables are renewed. Through a series of rules, the final selected solution is taken as the optimal solution.

Step 7: Setting termination conditions of TS. The maximum number of the iteration is $M_{p}.$ When the number of the iteration is greater than $M_{p}$ , the TS ends, and the current optimal solution is regarded as the final global optimization solution. 

## 5. Experimental Design and Results Analysis

The example validation includes two parts: in Section 5.1, the designed algorithm PSO-TS in this paper is tested utilizing the well-known data sets proposed by Barreto [35]; in Section 5.2, the effectiveness of the FLCOLRP model is proved by an example of a location-routing problem in post-earthquake relief deliveries. MATLAB R2017a is used to implement the PSO-TS algorithm, and all the experiments are evaluated on a PC with an Intel 2.3 GHz processor and 8DB RAM (Apple Inc., Cupertino, CA, USA).

### 5.1. Algorithm Experiment

In this section, we choose 10 cases (Gaspelle1-2, Christ50, Christ75, Christ100, Min27, Min134, Das88, Das150, Or170) from the typical database in LRP to test the applicability of the designed PSO-TS algorithm. Based on the previous literature [16,36], the parameters of PSO-TS are set as follows: The number of the population $N_{p}$ is 20, and the maximum number of the iteration $M_{p}$ is 500. In this study, the traditional PSO algorithm is compared with the proposed PSO-TS algorithm. Each of the following experiments is executed 20 times. The best value is recorded as the optimal result. Table 3 shows the number of candidate distribution centers (DCs), the number of demand points (DPs), as well as the detailed computational results of PSO and PSO-TS which include the number of open DCs and the total distance of dispatch.

We can easily see from Table 2 that, compared with the PSO algorithm, the number of open distribution centers, and the total dispatch distance of the PSO-TS algorithm, is 100% better excepting the total dispatch distance of Gaspelle2. Overall, they have a great improvement in the quality of the solution. Thus, the proposed PSO-TS algorithm in this paper is effective and competitive in tackling LRP.

### 5.2. Model Experiment

#### 5.2.1. Experimental Design

In this paper, the distribution data of a location-routing problem about emergency logistics, which are referred from the literature [22], is used to verify the FLCOLRP model. There are three candidate distribution centers and 20 demand points. Three types of vehicles are three each. The distance is the Euclidean distance in the test example. We suppose that between two nodes, the travel time of the vehicle is equal to the Euclidean distance. Table 4 illustrates the position, maximum capacity and construction costs of the candidate distribution centers. Table 5 shows the details about the demand points. Based on this information, the estimated demand can be calculated. The maximal weight, operation costs and longest distance of vehicles are displayed in Table 6. In order to be fair, the weights of the multi-objective function $\left(w_{1},\;w_{2},\;w_{3}\right)$ are initially set to 1/3. And we set the other parameters of the FLCOLRP model according to the former studies [22,31,37], which are shown in Table 7.

#### 5.2.2. Experimental Results

Urgent time, limited costs and environmental protection are all important in emergency logistics. From the overall emergency logistics’ point of view, the delivery time of relief supplies, the total costs for emergency logistics system and the carbon emissions need to be considered at the same time. Thus, we do the following experiments to achieve overall system optimization. Each experiment is implemented 20 times, and the numerical value with the best result is recorded.

First of all, we do the initial experiment when the $w_{1},\;w_{2}$ and $w_{3}$ have the same value 1/3. The experimental results show that the minimum delivery time is 1035.36, the minimum total cost is 508,982.92, and the minimum carbon dioxide emission is 488.79.

**Figure 3:**
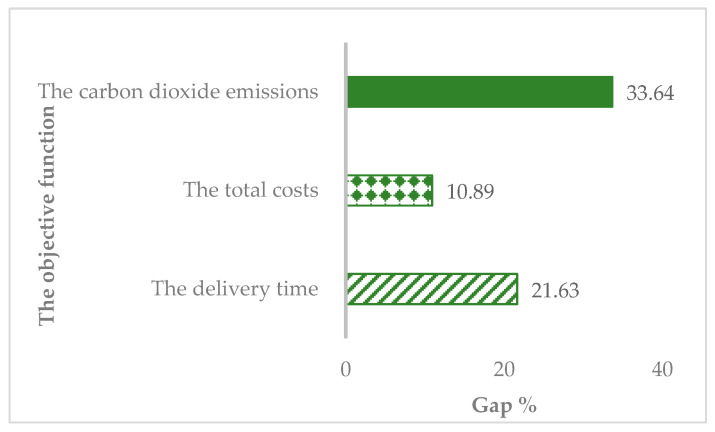
The difference between the initial experiment and the contrast experiment.

Next, in order to compare the results of the initial experiment, we calculate the minimum values of the delivery time F , the total costs C , and the carbon emissions $E_{co_{2}}$ respectively by setting different values of $w_{1},\;w_{2}$ and $w_{3}$ (0,1). Table 8 shows the contrast experimental results, including the specific values of $w_{1},\;w_{2}$ and $w_{3}$ . The difference between the results of the initial experiment and the results of the contrast experiment, shown in Figure 3, can be described by parameter Gap (Gap = (the results of the initial experiment- the results of the contrast experiment)/the results of the contrast experiment).

From the results in Figure 3, we can observe that the highest percentage of the difference belongs to the carbon emissions reaching 33.64%. The percentages of the difference between the delivery time and the difference between the total time are 20.01% and 10.89%, respectively. Obviously, there is still a certain gap between the minimum values of the initial experiment and the minimum values of the contrast experiment. For the best of the entire system, it is necessary to reduce this gap. It can be guessed that the minimum of the multi-objective function is related to the values of $w_{1},\;w_{2}$ and $w_{3}$ .

**Figure 4:**
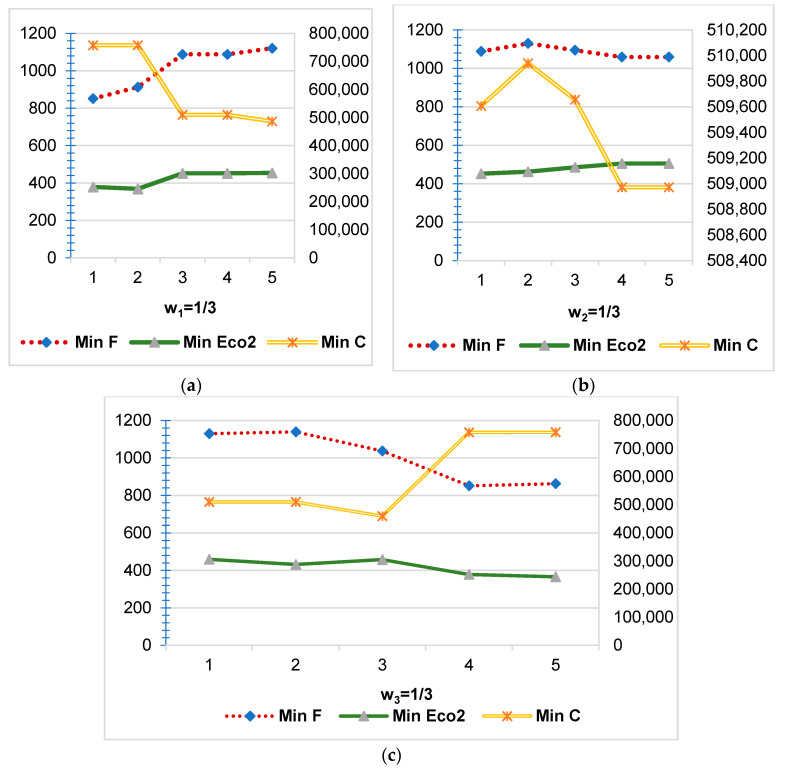
(**a**) The changing trends of the minimum of the delivery time, the total costs and the carbon emissions, when *w*_1_ = 1/3. (**b**) The changing trends of the minimum of the delivery time, the total costs and the carbon emissions, when *w*_2_ = 1/3. (**c**) The changing trends of the minimum of the delivery time, the total costs and the carbon emissions, when *w*_3_ = 1/3.

**Figure 5:**
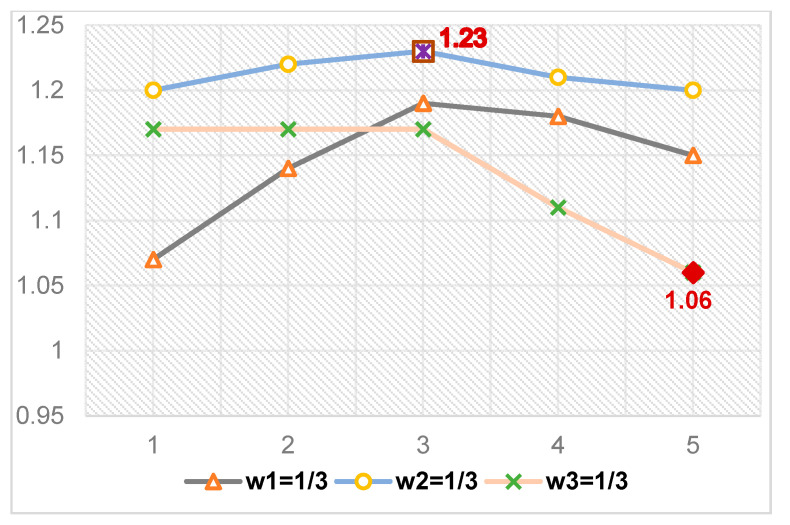
The changing trends of the optimal results of the total emergency logistics system.

Then, in order to study the relationship between the minimum of the multi-objective function ( MinF , MinC and $\mathrm{Min}E_{co_{2}}$ ) and the weight of $w_{1},\;w_{2}$ and $w_{3}$ , we do following tests thought setting series values of $w_{1},\;w_{2}$ and $w_{3}$ around the initial value 1/3. These tests consist of three types of trials ( $w_{1}=1/3,w_{2}=1/3,w_{3}=1/3$ ). The other weights for each type of trial are chosen from the five given values ( 1/12, 1/6, 5/12, 1/2, 7/12 ). The results of all experiments, including the values of $w_{1},\;w_{2}$ and $w_{3}$ , the minimum of multi-objective function ( MinF , MinC and $\mathrm{Min}E_{co_{2}}$ ) and the optimal value of the total emergency logistics system (Optimal) are shown in Table 9. In this study, the fitness function represents the optimal value of the total emergency logistics system. Figure 4 is composed of three-line charts ( $w_{1}=1/3,w_{2}=1/3,w_{3}=1/3$ ) describing the changing trends of the minimum of the delivery time, the total costs and the carbon emissions. The changing trends of the total optimal results of the emergency logistics system are shown in Figure 5.

From the results in Table 9, Figure 4 and Figure 5, we can observe the following findings:

1. The minimum delivery time is better when the weight of total costs becomes smaller, and the weight of the carbon emissions becomes bigger. When the weight of delivery time $w_{1}=1/3$ the minimum delivery time goes up gradually with the increase of the weight of total costs $w_{2}$ ; when the weight of total costs $w_{2}=1/3$ , the minimum rises initially, then goes up as the weight of delivery time $w_{1}$ go up; when the weight of carbon emissions $w_{3}=1/3$ , this figure goes up, then declines to the lowest value 851.21, finally rises slightly with the increase of $w_{1}$ . The range of the minimum delivery time is from 851.21 to 1139.40.
2. The minimum of total costs is better when the weight of delivery time and the weight of carbon emissions become smaller. When the weight of delivery time $w_{1}=1/3$ , the minimum of total costs levels out initially and declines afterwards with the increase of the weight of total cost $w_{2}$ ; when the weight of total costs $w_{2}=1/3$ , the minimum rises slightly, then drops dramatically, after that remains unchanged as the weight of delivery time $w_{1}$ goes up; when the weight of carbon emissions $w_{3}=1/3$ , this figure remains stable, then declines slowly, finally rises to the highest value 757,801.72 with the increase of $w_{1}$ . In brief, the minimum of total costs is between 757,801.72 and 458,997.82.
3. The minimum of carbon emissions is better when the weight of delivery time becomes bigger, and the weight of total costs becomes smaller. When the weight of delivery time $w_{1}=1/3$ , the minimum delivery time drops a little, then rises steadily with the increase of the weight of total costs $w_{2}$ ; when the weight of total costs $w_{2}=1/3$ , the minimum gradually increases reaching the highest value 504.65 as the weight of delivery time $w_{1}$ goes up; when the weight of carbon emissions $w_{3}=1/3$ , this figure rises and falls, finally decreases to the lowest value 504.65 with the increase of $w_{1}$ .
4. The changing of the weight of $w_{1},{\;w}_{2}$ and $w_{3}$ has an influence on the optimal result of the total emergency logistics system. From Table 9 and Figure 4, we can see that the optimal result rises, then declines when $w_{1}=1/3,$ and $w_{2}$ = 1/3 ; while this result remains stable, then drops when $w_{3}$ = 1/3 . Although all of them have a downward trend, the result of $w_{3}$ = 1/3 decreases more quickly than others. What’s more, the optimal result of the total emergency logistics system reaches the highest value 1.23 when $w_{2}=1/3,$ $w_{1}$ = 5/12, $w_{3}=1/4$ . It reaches the lowest value, 1.06, when $w_{3}=1/3,$ $w_{1}$ = 7/12, $w_{2}=1/12$ .

According to the above results, in order to optimize the entire emergency logistics system, we had to set $w_{1},\;w_{2},\;\;w_{3}$ as 7/12, 1/12, 1/3 , respectively, which means that delivery time is the most important, followed by environmental protection, and finally operation costs. It just coincides with the main characteristics of emergency logistics and low-carbon transportation. When $w_{1}$ = 7/12, $w_{2}=1/12,\;w_{3}=1/3$ , the results of the experiment show that three distribution centers are opened and seven vehicles are chosen in the test example. The specific selection of distribution centers and arrangement of vehicle routing can be seen in Table 10.

Finally, for the reason that the candidate distribution centers are all open in the example above, we add two new candidate distribution centers into the case for the sake of the application of the FLCOLRP model. The position of the two new candidate distribution centers is randomly chosen from the array 100×100 . The maximum capacity and construction costs of the candidate distribution centers are set based on the value of example. Table 11 displays the parameters of the new candidate distribution centers in detail. The experimental results about the service order of vehicle are demonstrated in Table 12.

As shown in Table 12, when the number of candidate distribution centers is five, the number of used vehicles is six (2,4,5,6,7,8) and the open distribution centers is four (DC1, DC2, DC3, DC4). Even though the number of candidate distribution centers is more, the results of the experiment are better. Besides, we still can obtain a good scheme of distribution. It indicates that the applicability of the FLCOLRP model in this study is good.

### 5.3. Analysis of Results

For the location-routing problem in emergency logistics, the multi-objective FLCOLRP model is built in this paper aiming at the optimization of the total emergency logistics system. By taking the minimum delivery time, total costs and carbon emissions as the objective functions, the routing plan that considers time, economic and environmental benefits can be obtained, thereby achieving “rapid and green” allocation scheme. Considering carbon emissions into the objective function is necessary for this environment with frequent natural disasters and bad climate. However, it is difficult to achieve the minimization of the three objective functions at the same time.

In this paper, by assigning weights to the three objective functions, we transform them into single objective function achieving the overall optimality of the emergency logistics system. The main summings-up are listed as follows:

1. The minimum of the multi-objective function is closely related to the value of weight ( $w_{1},w_{2},w_{3}$ . When $w_{2}=1/3,$ $w_{1}$ = 5/12, $w_{3}=1/4$ , the emergency logistics system obtains the overall optimality.
2. Though setting the different weight of objective functions and adding several candidate distribution centers, it is proved that the built FLCOLRP model in this study is applicable for LRP in emergency logistic.

Based on the above research, some constructive suggestions are put forward. From the perspective of the government emergency departments, firstly, they must strengthen people’s awareness of environmental protection, and improve education on how to take action after the disaster. Secondly, before the disaster, rescue measures have been planned, such as the construction of the rescue center, planning of the vehicle path, etc. As we all know, time is very urgent during the rescue, the cost of rescue is limited, and vehicles release carbon dioxide while delivering supplies. The government emergency departments can refer the results of this study to weigh the importance of the three to obtain the overall optimization of the emergency logistics system. Thirdly, carbon emissions during transportation should be emphasized. As a proverb says, a small leak will sink a great ship. The frequent occurrence of natural disasters is inseparable from climate change. From the perspective of the public, in order to minimize losses, it is important to learn to save the energy, which can extend the time as much as possible waiting for rescue. In addition, they must be educated that the greenhouse effect is one of the causes of frequent natural disasters, and take measures to protect the environment. For example, people can go out on foot or by public transportation, instead of private cars for the reduction of carbon emissions.

## 6. Conclusions

With the frequent occurrence of natural disasters, the lives and property of the people have already been caused by serious damage. However, natural disasters are closely related to environmental changes caused by human activities. The importance of environmental issues should be taken seriously. Thus, in the distribution of materials for emergency logistics, it is necessary to put carbon emissions into the overall optimal of emergency logistics for environmental protection and sustainable development. In this paper, aiming at the location–routing problem in the distribution process of relief supplies, the FLCOLRP model is established and solved by the hybrid PSO-TS algorithm. An example of a post-earthquake rescue is used to validate the established model. The minimum delivery time, total costs and carbon emissions are calculated respectively as the reference for subsequent experiments. Finally, the weight of the multi-objective function is ensured by a series of experiments to obtain the overall optimization. Based on the results, some suggestions are provided for the management department of emergency logistics and the citizen.

In this study, we obtain the overall optimization of emergency logistics through different settings for weight parameter values. However, we conduct comparative experiments around the weight value 1/3, due to experimental limitations. In future research, extensive and comprehensive comparative experiments are supposed to apply to the FLCOLRP model to get better overall optimization results. Besides, in a real emergency distribution environment, that emergency supplies are of various types can be considered into emergency logistics. What’s more, “embodied pollution” which can be considered into the emergency logistics system, is a new field about sustainable research.

## Figures and Tables

**Table 1:** Explanation of corresponding notations.

Notations	Explanation
$D_{c}$	Set of candidate distribution centers {i|i=1, …, C} .
$D_{o}$	Set of open distribution centers $D_{o}\subseteq D_{c}$ .
$N_{d}$	Set of demand points {i|i=C+1, C+2, …, C+D} .
$V_{h}$	Set of vehicles {h|h=1, 2, …, H} .
$P_{m}$	Set of sub-paths {m|m=1, 2, …, M} .
$t_{ij}$	Transportation time from node *I* to node *j*.
$C_{i}$	Construction and operation costs of the distribution center *i*.
$H_{h}$	Operation costs of the vehicle *h*.
$S_{1}$	Transportation costs of per unit distance.
$S_{2}$	Penalty costs of per unit unmet need.
${\left[ED_{i}\right]}^{o}$	Represents the most optimistic demand for demand point *i*.
${\left[ED_{i}\right]}^{l}$	Represents the most likely demand for demand point *i*.
${\left[ED_{i}\right]}^{p}$	Represents the most pessimistic demand for demand point *i*.
$\omega_{1}$	Weight coefficient of the most optimistic demand.
$\omega_{2}$	Weight coefficient of the most likely demand.
$\omega_{3}$	Weight coefficient of the most pessimistic demand.
$Q_{i}$	Demand for relief supplies of demand point *i.*
μ	Fuel consumption rate when the vehicle is full-load. Consumption Rate
$u_{0}$	Fuel consumption rate when the vehicle is no-load.
$M_{h}$	Maximal weight the vehicle *h* could carry.
$M_{ijh}$	Carried load of vehicle *h* from node *i* to node *j*.
τ	Conversion factor for carbon dioxide and fuel consumption.
$W_{i}$	Maximum capacity of candidate distribution center *i*.
$O_{h}$	Longest travel distance of the vehicle *h*.
$y_{i}$	$y_{i}=1$ represents the candidate distribution center *i* are employed.
	Otherwise, $y_{i}=0$ .
$x_{ij}^{h}$	$x_{ij}^{h}=1$ represents the vehicle *h* visit the node *j* from the node *i*.
	Otherwise, $\;x_{ij}^{h}=0$ .
$z_{mhi}^{c}$	$z_{mhi}^{c}=1$ represents the sub-path *m* of candidate distribution center c.
	includes node *i* served by the vehicle *h*. Otherwise, $z_{mhi}^{c}=0$ .

**Table 2:** Illustration of coding example.

Part 1	3	4	2	3	4	3	2	4
Part 2	1	**2**	**3**	**3**	4			
Part 3	10	7	5	8	12	9	6	11

**Table 3:** Computational results of PSO and PSO-TS.

Case	Number of DCs	Number of DPs	PSO		PSO-TS	
			Number of DCs	Total Distance	Number of DCs	Total Distance
**Gaspelle1**	5	21	3	544.57	1	545.01
**Gaspelle2**	5	22	2	892.69	1	898.07
**Christ50**	5	50	3	1462.01	3	1401.17
**Christ75**	10	75	7	2448.57	6	2316.46
**Christ100**	10	100	6	3027.12	6	2895.13
**Min27**	5	27	3	5744.55	1	5206.01
**Min134**	8	134	6	31,933.38	6	30,361.27
**Das88**	8	88	3	2411.84	3	2341.46
**Das150**	10	150	8	166,473.80	6	161,141.65
**Or117**	14	117	5	60,203.40	4	56,399.91

**Table 4:** Parameters of candidate distribution centers.

$\mathrm{Distribution}\;\mathrm{Centers}\;D_{i}$	X Coordinate	Y Coordinate	$\mathrm{Maximum}\;\mathrm{Capacity}\;W_{i}$	$\mathrm{Construction}\;\mathrm{Cos}\mathrm{ts}\;C_{i}\;(\mathrm{CNY})$
**1**	40	5	1500	200,000
**2**	70	60	2000	250,000
**3**	20	50	1800	300,000

**Table 5:** Parameters of demand points.

$\mathrm{Demand}\;\mathrm{Points}\;N_{i}$	X Coordinate	Y Coordinate	$\mathrm{Optimistic}\;\mathrm{Demand}\;{\left[ED_{i}\right]}^{o}$	$\mathrm{Likely}\;\mathrm{Demand}\;\;{\left[ED_{i}\right]}^{l}$	$\mathrm{Pessimistic}\;\mathrm{Demand}\;{\left[ED_{i}\right]}^{p}$
**4**	25	85	129	135	150
**5**	5	45	369	375	387
**6**	42	15	66	75	84
**7**	38	5	141	153	165
**8**	95	35	128	135	150
**9**	85	25	69	75	82
**10**	62	80	180	195	212
**11**	58	75	129	135	150
**12**	50	50	64	75	82
**13**	18	80	269	275	280
**14**	25	30	63	69	72
**15**	15	10	129	135	143
**16**	45	65	60	69	78
**17**	65	20	245	251	257
**18**	31	52	165	177	186
**19**	2	60	39	45	52
**20**	5	5	105	111	117
**21**	57	29	119	123	138
**22**	4	18	159	165	171
**23**	26	35	296	305	308

**Table 6:** Information about three types of vehicles.

$\mathrm{Number}\;\mathrm{of}\;\mathrm{Vehicles}\;V_{h}$	$\mathrm{Maximal}\;\mathrm{Weight}\;M_{h}$	$\mathrm{Operation}\;\mathrm{Cos}\mathrm{ts}\;H_{h}\;(\mathrm{CNY})$	$\mathrm{Longest}\;\mathrm{Distance}\;O_{h}\;(\mathrm{km})$
**1–3**	100	500	350
**4–6**	150	700	360
**7–9**	200	900	370

**Table 7:** Parameters related to the objective function.

Parameters	Value
$S_{1}$	8 CNY/km
$S_{2}$	150 CNY/kg
μ	0.165 L/km
$u_{0}$	0.377 L/km
τ	2.63 kg/L

**Table 8:** The results of the contrast experiment.

Value	MinF (min)	MinC (NCY)	$MinE_{co_{2}}$ (kg)
$w_{1}=1,$ $\;w_{2}$ = $w_{3}=0$	851.21	-	-
$w_{2}=1,$ $\;w_{1}$ = $w_{3}=0$	-	458,997.82	-
$w_{3}=1,$ $\;w_{1}$ = $w_{2}=0$	-	-	365.76

**Table 9:** The results of the three types of experiments ( $w_{1}=1/3,w_{2}=1/3,w_{3}=1/3$ ).

Value			MinF (min)	MinC (NCY)	$MinE_{co_{2}}\;(\mathrm{kg})$	Optimal
$w_{1}=1/3$	$w_{2}$ = 1/12, $w_{3}=7/12$	1	851.21	757,509.68	378.39	1.07
	$w_{2}$ = 1/6, $w_{3}=1/2$	2	913.09	757,804.73	368.25	1.14
	$w_{2}$ = 5/12, $w_{3}=1/4$	3	1088.24	509,605.92	451.42	1.19
	$w_{2}$ = 1/2, $w_{3}=1/6$	4	1088.24	509,605.92	451.42	1.18
	$w_{2}$ = 7/12, $w_{3}=1/12$	5	1120.93	486,267.49	453.73	1.15
$w_{2}=1/3$	$w_{1}$ = 1/12, $w_{3}=7/12$	1	1097.92	574,483.42	435.06	1.20
	$w_{1}$ = 1/6, $w_{3}=1/2$	2	1129.94	509,939.58	461.62	1.22
	$w_{1}$ = 5/12, $w_{3}=1/4$	3	1094.38	509,655.09	484.96	1.23
	$w_{1}$ = 1/2, $w_{3}=1/6$	4	1058.95	508,971.64	504.65	1.21
	$w_{1}$ = 7/12, $w_{3}=1/12$	5	1058.95	508,971.64	504.65	1.20
$w_{3}=1/3$	$w_{1}$ = 1/12, $w_{2}=7/12$	1	1129.57	509,536.58	458.94	1.17
	$w_{1}$ = 1/6, $w_{2}=1/2$	2	1139.40	509,615.18	431.38	1.17
	$w_{1}$ = 5/12, $w_{2}=1/4$	3	1037.23	458,997.82	457.41	1.17
	$w_{1}$ = 1/2, $w_{2}=1/6$	4	851.21	757,509.68	378.39	1.11
	$w_{1}$ = 7/12, $w_{2}=1/12$	5	862.71	757,801.72	365.76	1.06

**Table 10:** The service order of vehicles ( $w_{1}=7/12,w_{2}=1/12,w_{3}=1/3$ ).

Number of Vehicle	Service Order	Number of Vehicle	Service Order
**2**	DC3-11-DC2	**7**	DC3-18-23-DC3-DC1-6-DC1
**3**	DC3-4-5-19-DC3-DC2-10-DC2	**8**	DC3-20-DC1-DC2-8-16-13-DC3-DC2-9-DC2
**4**	DC3-14-DC3	**9**	DC1-15-DC1-DC3-12-DC2
**6**	DC1-7-22-17-21-DC1		

**Table 11:** Parameters of two new candidate distribution centers.

$\mathrm{Distribution}\;\mathrm{Centers}\;D_{i}$	X Coordinate	Y Coordinate	$\mathrm{Maximum}\;\mathrm{Capacity}\;W_{i}$	$\mathrm{Construction}\;\mathrm{Cos}\mathrm{ts}\;C_{i}\;(\mathrm{CNY})$
4	5	20	1900	240,000
5	90	80	1500	300,000

**Table 12:** The service order of vehicles ( $w_{1}=7/12,w_{2}=1/12,w_{3}=1/3$ ).

Number of Vehicle	Service Order	Number of Vehicle	Service Order
**2**	DC4-22-DC4	**6**	DC1-9-DC1
**4**	DC3-6-DC4	**7**	DC3-7-13-16-DC3-DC2-10-19-12-14-DC2
**5**	DC4-25-DC3-DC1-8-11-20-DC3-DC1-17-23-DC1	8	DC3-21-24-DC4-DC3-15-18-DC2
